# Comparative analysis of cytokine/chemokine regulatory networks in patients with hippocampal sclerosis (HS) and focal cortical dysplasia (FCD)

**DOI:** 10.1038/s41598-017-16041-w

**Published:** 2017-11-21

**Authors:** Arpna Srivastava, Aparna Banerjee Dixit, Debasmita Paul, Manjari Tripathi, Chitra Sarkar, P. Sarat Chandra, Jyotirmoy Banerjee

**Affiliations:** 1 Centre of Excellence for Epilepsy, a joint collaboration of NBRC & AIIMS, New Delhi, India; 2 Department of Neurosurgery, All India Institute of Medical Sciences, New Delhi, India; 3 Dr. B.R. Ambedkar Centre For Biomedical Research, University of Delhi, Delhi, India; 4 Department of Neurology, All India Institute of Medical Sciences, New Delhi, India; 5 Department of Pathology, All India Institute of Medical Sciences, New Delhi, India; 6 Department of Biophysics, All India Institute of Medical Sciences, New Delhi, India

## Abstract

Experimental and clinical evidence have demonstrated aberrant expression of cytokines/chemokines and their receptors in patients with hippocampal sclerosis (HS) and focal cortical dysplasia (FCD). However, there is limited information regarding the modulation of cytokine/chemokine-regulatory networks, suggesting contribution of miRNAs and downstream transcription factors/receptors in these pathologies. Hence, we studied the levels of multiple inflammatory mediators (IL1β, IL1Ra, IL6, IL10, CCL3, CCL4, TNFα and VEGF) along with transcriptional changes of nine related miRNAs and mRNA levels of downstream effectors of significantly altered cytokines/chemokines in brain tissues obtained from patients with HS (n = 26) and FCD (n = 26). Up regulation of IL1β, IL6, CCL3, CCL4, STAT-3, C-JUN and CCR5, and down regulation of IL 10 were observed in both HS and FCD cases (p < 0.05). CCR5 was significantly up regulated in FCD as compared to HS (p < 0.001). Both, HS and FCD presented decreased miR-223-3p, miR-21-5p, miR-204-5p and let-7a-5p and increased miR-155-5p expression (p < 0.05). As compared to HS, miR-204-5p (upstream to CCR5 and IL1β) and miR-195-5p (upstream to CCL4) were significantly decreased in FCD patients (p < 0.01). Our results suggest differential alteration of cytokine/chemokine regulatory networks in HS and FCD and provide a rationale for developing pathology specific therapy.

## Introduction

Focal cortical dysplasia (FCD) caused by the malformations of cortical development (MCDs) and hippocampal sclerosis (HS) are the two most frequent drug resistant epilepsy (DRE) pathologies which constitutes about fifty percent of all surgical pathology of epilepsy^1^. FCD and HS are network-level disorders involving alterations in several cellular processes and multiple cell signalling pathways^2,3^. Brain inflammation is an intrinsic feature of the hyperexcitable pathologic brain tissue in DREs of differing etiology^4^. There is robust evidence for an activated immune response in non-CD pathology like HS. The activation of inflammatory pathways in human HS is supported by gene expression analysis^2^. An elevated level of interleukin 6 (IL6), IL1β, chemokines (Chemokine (C-C motif) ligand) (CCL3, CCL4) are reported in the cerebrospinal fluid and brain tissues of HS patients^5,6^. In experimental models, up regulation of the IL1β, IL6, IL1Ra, tumor necrosis factor alpha (TNFα) and tumor growth factor beta (TGFβ1) are reported^7^. Moreover, a direct pro-convulsive effect of IL1β is demonstrated^8^. These observations suggest the existence of a feedback loop between the pro-inflammatory cytokine/chemokine systems, which may be critical for the propagation of the inflammatory response in human TLE with HS^9^.

Induced inflammatory response in FCD is supported by the activation of IL6 and IL1β signalling pathways, induction of the chemokines, microglial reactivity, as well as blood brain barrier (BBB) breakdown^9,10^. There is also evidence of activation of the plasminogen, the toll like receptor (TLR) and the vascular endothelial growth factor (VEGF)-mediated signalling contributing to glial activation and the associated inflammatory reactions^11^.

There is a growing body of evidence supporting that the regulation of inflammatory response is a very complex process involving coordinated participation of multiple regulation systems, such as an integrated network of microRNAs (miRNAs) and transcription factors/receptors^12^. Little is known regarding the postulated cytokine/chemokine regulatory network in HS and FCD; however, inflammation related mediators have been implicated in a number of studies. The aim of the present study was to analyze the cytokine-chemokine regulatory networks in the brain tissues resected from patients with HS and FCD.

To the best of our knowledge, direct, simultaneous quantification of multiple inflammation-related mediators and its regulators in resected tissues of human HS, FCD, and non-epileptic brain tissue has not been previously accomplished. Therefore, we measured the level of eight inflammatory mediators (IL1β, IL1Ra, IL6, IL10, CCL3(MIP1α), CCL4(MIP1β), TNFα and VEGF) and investigated the gene expression of nine inflammation-related miRNAs (miR-106a-5p, miR-223a-3p, miR-21-5p, miR-195-5p, miR-204-5p, miR-203-3p, miR-155-5p, let-7a-5p and let-7c-5p) and four downstream effectors (STAT-3, C-JUN, ICER and CCR5) in brain tissues obtained from fifty two epilepsy patients (twenty six HS and twenty six FCD) and twenty two non-epileptic control subjects using multiplex immunoassay and quantitative RT-PCR respectively. The inflammatory mediators and the downstream effectors were selected based on previous literatureand contribution in inflammatory processes^13–18^ whereas miRNAs were identified through miRTarBase^19^.

## Results

### Characteristics of individuals

A total of twenty six HS (age range-6 to 43 yrs) and twenty six FCD (age range-4 to 51 yrs) patients were included in this study. For non-epileptic control (age range- 3 to 63 yrs), cortical tissues were obtained from the margins of tumor from the patients who underwent curative surgery because of brain tumors (twenty two) having no seizures. For the cytokine/chemokine assay, ten HS (seven male and three female), ten FCD (six male and four female) and eight controls (six male and two female) were included. Subsequently, we used surgically resected tissues from ten HS (six male and four female), ten FCD (five male and five female) and eight controls (five male and three female) for downstream gene expression analysis whereas six HS (three male and three female), six FCD (four male and two female) and six control (four male and two female) were used for micoRNA expression studies. The detailed clinical characteristics of individuals were listed in Table 1.

**Table 1:** Clinical data of patients and controls.

Patient/Control	Age(Years)/Sex	Pathology	AEDs
H1	34/M	HS	Levetiracetam, Carbamazepine, Clobazam
H2	37/F	HS	Sodium valproate, Clobazam, Lamotrigene
H3	28/F	HS	Oxcarbazepine, Levetiracetam, Clobazam
H4	14/M	HS	Levetiracetam, Sodium valproate, Clobazam, Lamotrigene
H5	18/M	HS	Clonazepam, Carbamazepine, Sodium valporate
H6	24/F	HS	Oxcarbazepine, Levetiracetam, Clobazam
H7	15/M	HS	Phenytoin, Clobazam
H8	31/M	HS	Clonazepam, Carbamazepine, Sodium valporate
H9	24/M	HS	Levetiracetam, Sodium valproate, Clobazam, Lamotrigene
H10	20/M	HS	Sodium valproate, Clobazam
H11	35/M	HS	Levetiracetam, Carbamazepine, Clobazam
H12	15/M	HS	Clobazam, Sodium valproate, Phenobarbital
H13	36/F	HS	Oxcarbazepine, Levetiracetam, Clobazam
H14	27/F	HS	Levetiracetam, Clobazam
H15	6/M	HS	Clobazam, Levetiracetam, Phenytoin, Sodium valproate, Clonazepam
H16	6/M	HS	Clobazam, Sodium valproate, Phenobarbital
H17	15/M	HS	Lacosamide, Clobazam, Lamotrigene, Levetiracetam
H18	23/F	HS	Oxcarbazepine, Levetiracetam, Clobazam
H19	9/M	HS	Phenytoin, Carbamazepine, Risperidonesyp
H20	27/F	HS	Lacosamide, Clobazam, Levetiracetam
H21	36/F	HS	Lacosamide, Clobazam, Lamotrigene, Levetiracetam
H22	42/F	HS	Lacosamide, Levetiracetam
H23	22/M	HS	Clobazam, Lacosamide, Olanzapine, Fluoxetine
H24	43/M	HS	Clobazam, Levetiracetam, Carbamazepine
H25	20/F	HS	Clobazam, Levetiracetam, Sodium valporate
H26	24/M	HS	Lamotrigene, Levetiracetam, Oxcarbazepine
F1	5/F	FCD TYPE IIB	Lacosamide, Clobazam, Levetiracetam
F2	6/M	FCD TYPE IIA	Clobazam, Levetiracetam, Carbamazepine
F3	23/M	FCD TYPE IIB	Clobazam, Levetiracetam, Sodium valporate
F4	4/F	FCD TYPE IIA	Lacosamide, Clobazam, Levetiracetam
F5	6/M	FCD TYPE IIA	Oxcarbazepine, Sodium valporate
F6	7/M	FCD TYPE IIB	LevetiracetamOxcarbazepine, Phenytoin
F7	22/M	FCD TYPE IIA	Clobazam, Carbamazepine, Topiramet
F8	13/M	FCD TYPE IIA	Clobazam, Levetiracetam, Sodium valporate
F9	24/F	FCD TYPE IIA	Lacosamide, Clobazam, Levetiracetam
F10	24/F	FCD TYPE IIB	Clobazam, Levetiracetam, Carbamazepine
F11	12/F	FCD TYPE IIA	Levetiracetam, Clobazam, Phenytoin
F12	22/M	FCD TYPE IIB	Clobazam, Levetiracetam, Sodium valporate
F13	1/M	FCD TYPE IIA	Clonazepam, sodium valproate, Levetiracetam, Vigabartin, Zonisamide
F14	14/F	FCD TYPE IIB	Lacosamide, Clobazam, Levetiracetam
F15	16/F	FCD TYPE IIB	Clobazam, Levetiracetam, Carbamazepine
F16	51/F	FCD TYPE IIA	Clobazam, Levetiracetam, Topiramet
F17	14/M	FCD TYPE IIA	Lacosamide, Clobazam, Levetiracetam, Oxcarbazepine
F18	14/M	FCD TYPE IIB	Clobazam, Levetiracetam, Carbamazepine
F19	6/M	FCD TYPE IIA	Clobazam, Levetiracetam, Phenytoin, Sodium valporate
F20	41/F	FCD TYPE IIA	Levetiracetam, Oxcarbazepine, Phenytoin
F21	22/M	FCD TYPE IIA	Clobazam, Levetiracetam, Carbamazepine
F22	9/M	FCD TYPE IIA	Lacosamide, Clobazam, Levetiracetam
F23	19/M	FCD TYPE IIA	Clobazam, Lamotrigene
F24	15/F	FCD TYPE IIA	Clobazam, Levetiracetam, Carbamazepine
F25	16/F	FCD TYPE IIB	Clobazam, Levetiracetam, Carbamazepine
F26	19/M	FCD TYPE IIA	Clobazam, Levetiracetam, Topiramet
C1	45/F	Right temporopartial high grade Glioma	NA
C2	32/M	Right frontal low grade glioma	NA
C3	30/M	Temporal-occipital tumour	NA
C4	30/M	Right temporopartial glioma	NA
C5	60/M	Right insular glioma	NA
C6	50/F	Meningioma	NA
C7	38/M	Temporooccipitaltumor	NA
C8	30/M	Right frontal glioma	NA
C9	54/F	Left temporoparietal high grade glioma	NA
C10	22/M	Intraventricularneurocytoma	NA
C11	57/M	Meningioma	NA
C12	48/M	Left frontal recurrent oligodendrioglioma	NA
C13	30/F	Meningioma	NA
C14	35/M	Right temporoparietal, glioma	NA
C15	3/M	Left thalamic glioma	NA
C16	30/F	Left insular glioma	NA
C17	53/M	Posterior frontal glioma	NA
C18	63/F	Meningioma	NA
C19	40/M	Petroclival meningioma	NA
C20	34/M	Right frontal low grade glioma	NA
C21	56/F	Meningioma	NA
C22	38/M	Left insular glioma	NA

COD- Cause of death, H- HS, F- FCD, C- Control, AEDs-Anti-epileptic drugs.

### Protein expression of the inflammatory mediators in HS and FCD patients

Up regulation of IL1β, IL6, CCL3 and CCL4 and down regulation of IL 10 were observed in both HS and FCD patients as compared to non-epileptic controls (Table 2, p < 0.05). Significant up regulation of IL6 (HS p < 0.01), FCD p < 0.001), IL1β (HS: p < 0.005, FCD p < 0.05), CCL3 (HS: p < 0.005, FCD p < 0.001) and CCL4 (HS: p < 0.05, FCD p < 0.001) were observed in both HS and FCD as compared to the non-epileptic controls (Table 2). IL 10 was significantly down regulated in HS and FCD (HS: p < 0.01, FCD: p < 0.01) as compared to the non-epileptic control. None of these cytokines and chemokines significantly differed among the HS and FCD patient groups (p > 0.05; Table 2). TNFα, VEGF and IL1Ra did not show any significant change between groups (Table 2).

**Table 2:** Protein levels of inflammatory molecules (pg/ml) in the study and control groups.

Protein	Group 1 (Control)	Group 2 (HS)	Group 3 (FCD)	p-value		
				Group 1 vs Group 2	Group 1 vs Group 3	Group 2 vs Group 3
IL1β	1.795 (1.05–2.43)	4.065 (1.57–12.31)	2.680(1.88–10.72)	p = 0.003	p = 0.020	p = 1.00
IL1Ra	30.10 (19.74–96.98)	30.76 (3.60–81.69)	44.72 (4.98–81.69)	p > 0.05	p > 0.05	p > 0.05
IL6	0.96 (0.73–1.29)	2.495 (0.96–3.75)	2.62(1.12–5.61)	p = 0.008	p < 0.001	p = 1.00
IL10	0.77 (0.11–0.92)	0.16 (0.01–0.66)	0.15 (0.04–0.70)	p = 0.009	p = 0.008	p = 1.00
CCL3	10.28 (8.05–13.56)	45.44 (16.07–345.51)	72.11 (15.52–511.22)	p = 0.001	p < 0.001	p = 1.00
CCL4	4.095 (2.33–6.16)	7.87 (6.13–22.54)	25.72 (4.53–41.68)	p = 0.018	p < 0.001	p = 0.795
TNFα	1.05 (0.5–3.09)	1.89 (0.39–5.38)	1.470 (0.72–5.61)	p > 0.05	p > 0.05	p > 0.05
VEGF	46.28 (40.87–56.85)	48.29 (21.99–251.49)	50.36 (32.67–246.49)	p > 0.05	p > 0.05	p > 0.05

### Changes in the gene expression profiles

**Figure 1:**
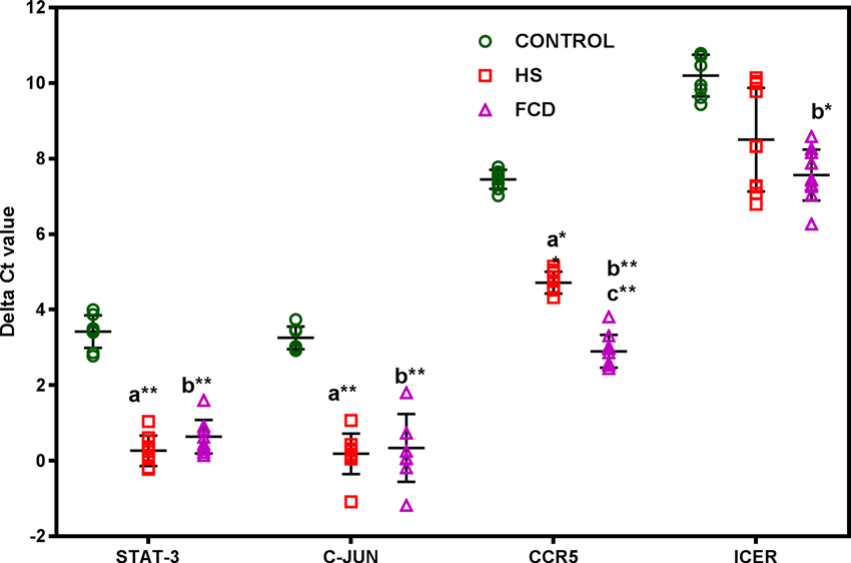
The relative amount of mRNA transcripts in patients analyzed using quantitative real time PCR. Values are represented as delta cycle threshold (ΔCt). Error bar is mean ± SD based on ten patients from each group and eight control samples, and each sample is analyzed in triplicates. Mean increase in transcripts levels are statistically significant (One-way ANOVA followed by the Tukeys’ post hoc test; *p < 0.05; **p < 0.01; a = Control vs HS, b = Control vs FCD; c = HS vs FCD).

A significant increase in STAT-3 expression was observed in HS (p < 0.001, 8.89 fold; ranges from 6.72 to 11.77 fold) and FCD (p < 0.001; 6.88 fold; ranges from 5.06 to 9.35 fold) as compared to non-epileptic control (Fig. 1). C-JUN expression was found to be up regulated in both, HS (p < 0.001; 8.40 fold; ranges from 5.79 to 12.18 fold) and FCD (p < 0.001; 7.52 fold; ranges from 4.04 to 14.02 fold) as compared to non-epileptic controls (Fig. 1). The level of the ICER transcript was significantly changed only in FCD patients (p < 0.05; 6.21 fold; ranges from 3.88 to 9.92 fold) as compared to the non-epileptic control group (Fig. 1). We did not observe any significant changes in the ICER level in HS when compared to non-epileptic control as well as FCD (p > 0.05, Fig. 1). CCR5 expression level was found to be significantly altered in HS (p < 0.001; 6.39 fold increase; ranges from 5.22 to 8.28 fold) and FCD (p < 0.001; 23.49 fold increase;ranges from 17.39 to 31.72 fold; Fig. 1) when compared with non-epileptic control. Significant increase of CCR5 (p < 0.001; 3.67 fold; ranges from 3.32 to 3.83 fold; Fig. 1) in FCD has been demonstrated as compared to HS. However, there was no significant difference observed in STAT-3 and C-JUN level between HS and FCD patients (p > 0.05; Fig. 1).

### Changes in the expression pattern of miRNAs

**Figure 2:**
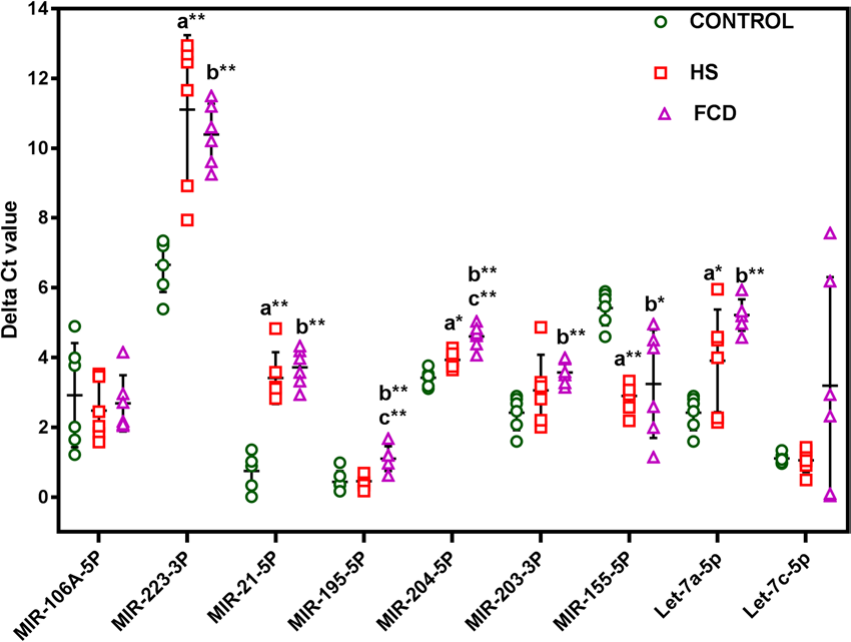
Differential expression of miRNAs in epilepsy patients. Values are represented as delta cycle threshold (ΔCt). Error bar is mean ± SD based on six patients from each group and six control samples, and each sample is analyzed in triplicates. Mean increase in transcripts levels are statistically significant (One-way ANOVA followed by the Tukeys’ post hoc test; *p < 0.05; **p < 0.01; a = Control vs HS, b = Control vs FCD; c = HS vs FCD).

HS patients presented decreased miR-223-3p (p < 0.001; upstream to IL6, CCL3 −21.75 fold decrease;ranges from −12.63 to −37.48 fold), miR-21-5p (p < 0.001; upstream to IL 1β, STAT-3, −6.34 fold decrease; ranges from −3.80 to −10.59 fold), and miR-204-5p (p < 0.05, upstream to IL 1β, CCR5, −1.43 fold; ranges from-1.21 to −1.68 fold;) and let-7a-5p (p < 0.05, upstream to IL 6; −2.80 fold; ranges from −1.01 fold to −7.77 fold) and increased miR-155-5p (p < 0.01; upstream to IL6, STAT3, C-JUN, 5.72 fold increase; ranges from 4.19 to 7.80) compared with non-epileptic controls (Fig. 2).

FCD patients presented decreased miR-223-3p (p < 0.01; upstream to IL6, CCL3, −13.29 fold decrease;ranges from −7.23 to −24.42), miR-21-5p (p < 0.001;(upstream to IL 1β, STAT-3, −7.85 fold decrease;ranges from −5.40 to −11.40 fold), miR-204-5p (p < 0.001; upstream to IL 1β, CCR5, −2.28 fold decrease;ranges from −1.81 to − 2.88- fold), miR-195-5p (p < 0.005; upstream to CCL4, −1.58 fold decrease; ranges from −1.24 to −2.02 fold), miR-203-3p (p < 0.005; upstream to IL6, C-JUN, CCR5, −2.22 fold decrease; ranges from-1.75 fold to −2.83 fold) and let-7a-5p (p < 0.001; upstream to IL6,−6.97 fold decrease; ranges −5.09 to −9.54 fold) and increased miR-155-5p (p < 0.05; upstream to IL6, STAT3, C-JUN,4.52 fold increase; ranges from 1.54 fold to 13.23 fold increase), compared with non-epileptic controls (Fig. 2).

Expression of miR-204-5p (p < 0.01; upstream to IL1β and CCR5, −1.59 fold decrease; ranges from −1.48 fold to −1.71 fold) and miR-195-5p (p < 0.01; upstream to CCL4, −1.60 fold decrease; ranges from −1.44 to −1.78 fold decrease) only found to be decreased in FCD when compared with HS (Fig. 2). Rest showed no significant difference between HS and FCD (Fig. 2). miR-195-5p (upstream to CCL4) and miR-203-3p (upstream to IL6, C-JUN, and CCR5) were found to be significantly decreased only in FCD, not in HS (Fig. 2). No significant difference was observed in the levels of miR-106a-5p (upstream to IL10, STAT3) and let-7c-5p (upstream to IL 6, IL 10, and ICER) between groups (Fig. 2).

### Analysis of cytokine/chemokine-receptor network

**Figure 3:**
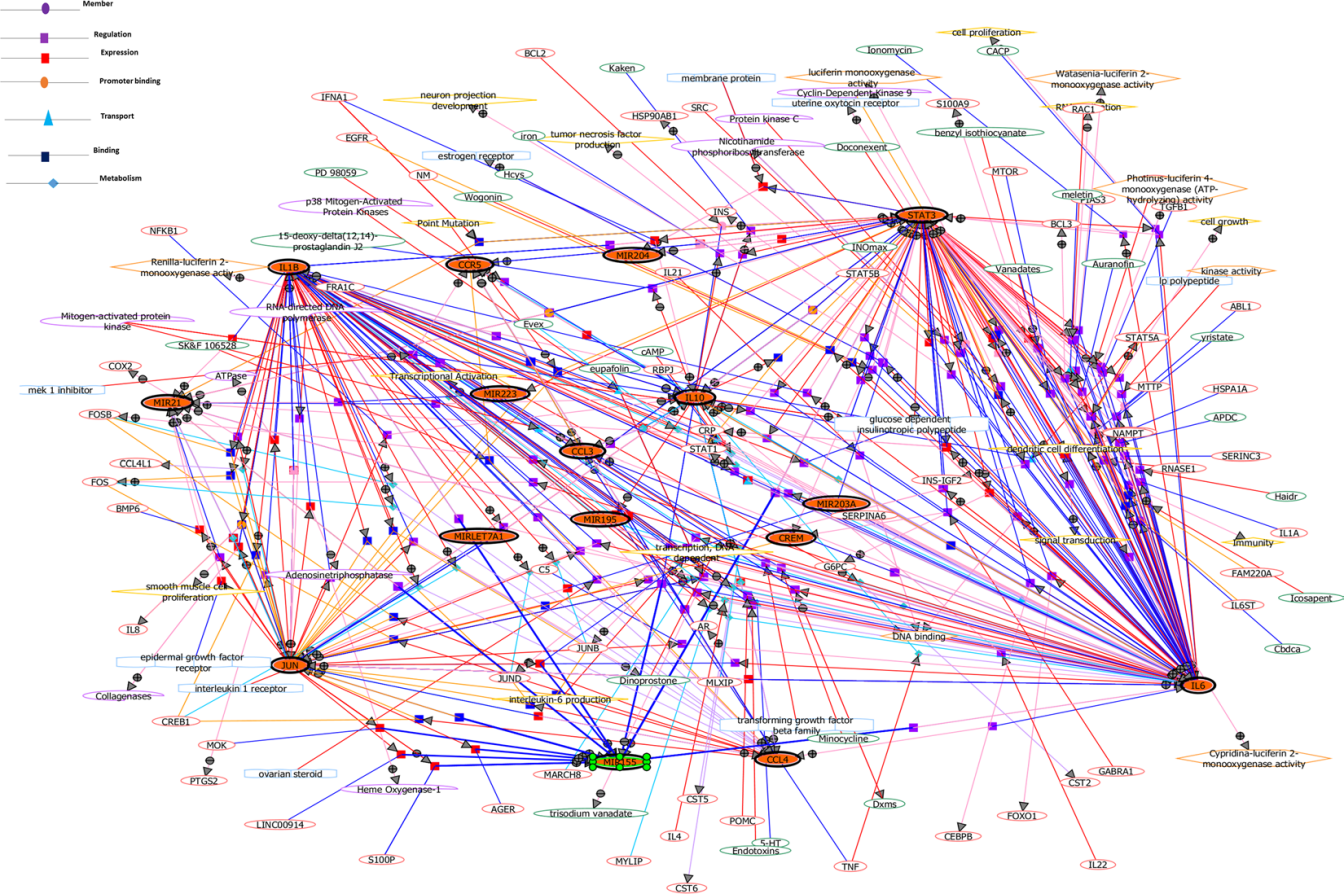
Gene network analysis showing associations between significantly modulated studied cytokines/chemokines, microRNAs and its downstream effectors. Genes found modulated in our study are shown in filled circles. Different coloured edges with arrows shows the direction of interactions. Circles with + and − symbols represents positive and negative regulations. Symbols (small squares, triangles and circles) shows various modes of regulations like, binding, expression, transport, promoter binding etc.

**Figure 4:**
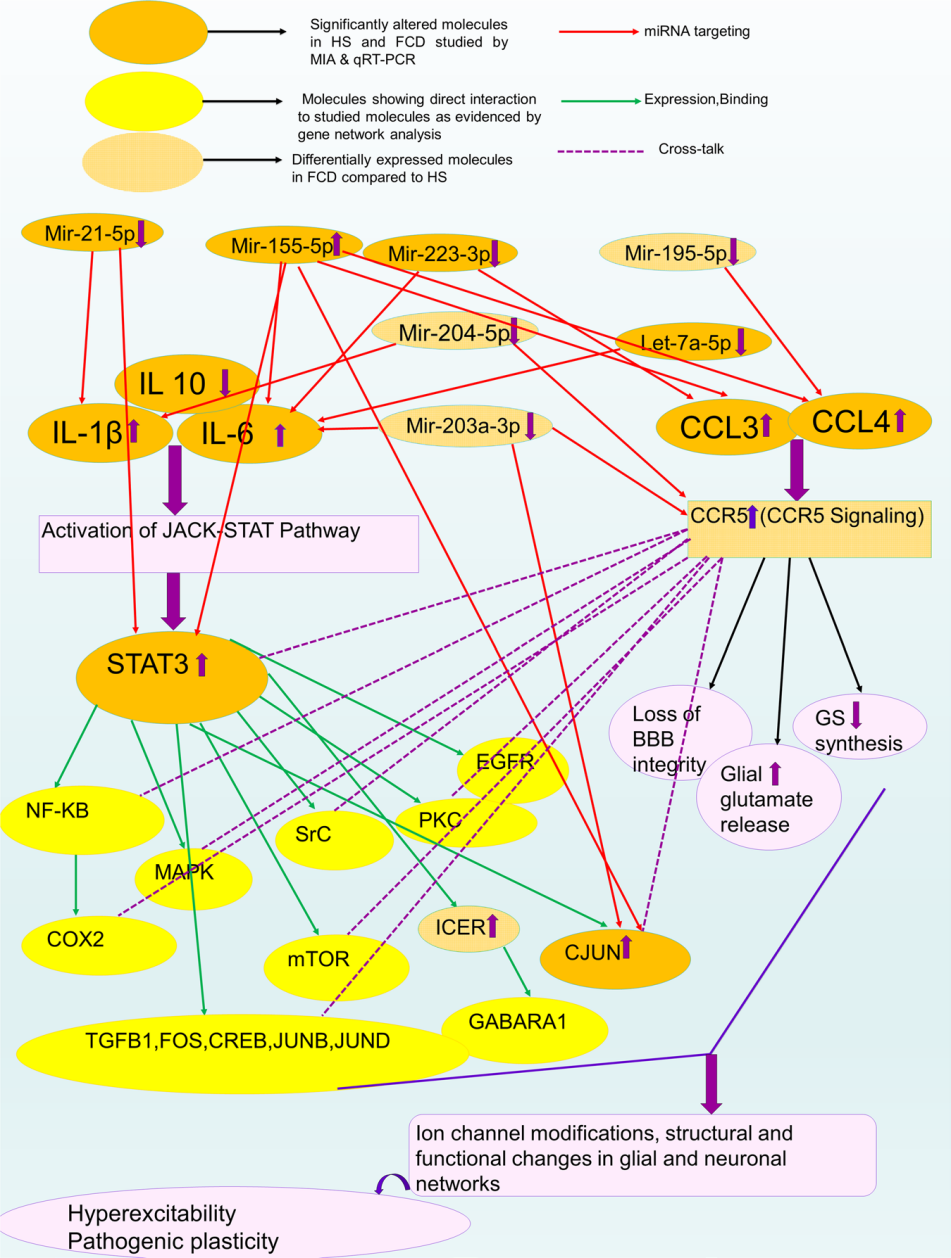
Predicted network of significantly altered inflammatory mediators, downstream transcription factors/receptors and microRNAs based on MIA, qPCR, gene network analysis and previous literatures (Ref. no. −2, 13–19, 41, 43).

Gene network analysis was performed to look at the associations of the significantly altered cytokines/chemokines, upstream microRNAs and the downstream effectors. Gene network analysis revealed some molecules of more connectivity like IL6, IL1β, STAT3, C-JUN, IL10, CCR5, miR-155 and miR-21, constituting major hubs. These molecules were further showing interaction with other molecules previously shown to be associated with epileptogenesislikeNF-κB, COX-2, FOS, CREB, GABARA1, PKC, TGFβ1, BCL-2, SrC, EGFR, MAPK, mTORetc (Fig. 3). As these molecules are already implicated in epileptogenesis, this analysis further confirms the complexity of the DRE involving dysregulation of several pathways(Figs 3 and 4).

## Discussion

This is the first comparative study demonstrating deregulated cytokine/chemokine regulatory networks in the HS and FCD. Although, a number of altered molecules are common in both pathologies, but differential expression of CCR5, miR-204 and miR-195 are demonstrated in FCD. Taking all these in consideration with analysis of the similarities and differences among the networks, we are speculating that altered cytokine/chemokine-receptor network might contribute differentially in the two drug resistant pathologies with the chemokine signalling being more prevalent in FCD. The tumor periphery cortical tissues used for non-epileptic controls were from individuals with brain tumors and who might have fairly pronounced abnormalities in their cytokines and chemokinesin the tumorous tissues or in the peripheral blood^20–22^. To avoid possible effects of an inflammatory response, we used samples from the periphery of thetumor as non-epileptic controls. Furthermore, neuropathologists reviewed the non-epileptic control cases, and both gross and microscopic examinations revealed no abnormalities and tissues used as non-epileptic controls were tumor free. Alterations in cytokines and chemokines have been reported in tumor tissues and peripheral blood, but there are no reports showing chemokine/cytokinealterations inthe tumor periphery tissue. Owing to different limitations associated with autopsy, trauma and tumor periphery tissues, we preferred to use histopathologically normal tumor periphery tissues asnon-epileptic controlsfor this study, as suchnon-epileptic controls have been used previously in other studies^2,23–25^.

### Cytokines-transcription factor-miRNA regulatory network

Similar to previous studies, significant up regulation of the cytokines IL1β and IL6 and down regulation of IL10 wasobserved in brain tissues of both HS and FCD patients^5,26–29^. Few studies reporting IL1Ra expression have shown contradictory results. IL1Ra is a naturally occurring antagonist of IL-1 receptor type 1, which acts by limiting IL1β-mediated actions^30^. Lack of IL1Ra modulation in our study might contributeto increased pro-inflammatory state of the brain tissues in both HS and FCD. IL10 suppresses the production of pro-inflammatory cytokines and chemokines, thus down regulation of IL10 might confer decreased anti-inflammatory state^5,27^. Unaltered TNFα levels in our study is comparable whereas no change in VEGF is contradictory to previous reports in HS patients^5,27^, demanding further investigations for more conclusive findings.

IL1β & IL6 are found to have pro-epileptogenic properties in animal models for epilepsy, particularly, IL1β, which affects neuronal Ca^2+^ influx through NMDA dependent signalling^8,29^. Thus, up regulation of IL1β & IL6 may implicate a pro-inflammatory state whereas down regulation of IL10 and unaltered IL1Ra in our study could be attributed to deregulation of the pathway, thus failing to counteract the pro-inflammatory state.

STAT proteins are principal mediators of cytokine receptor signalling and a number of STAT3 target genes have been found to be involved in the process of epileptogenesis^13–15^. Activation of IL6 and IL1β signalling via JAK-STAT pathway has been demonstrated inHS and FCD patients^13–15^. C-JUN, andJNK are found to be hyperactivated and antiepileptic action of JNK inhibition has been reported in animal model of epilepsy^17^. Hu *et al*. (2008) demonstrated that increase in ICER mRNA following status epilepticus might have a role in suppressing the severity of epilepsy in animal models but no such reports are available in epilepsy patients^16^. We observed increased STAT-3 andC-JUN expression in surgically resected tissues from both HS and FCD patients whereas ICER up regulation was only observed in FCD. This suggests up regulation of IL1β & IL6 signalling pathways might be mediated via JAK/STAT signalling in both HS and FCD but the downstream effectors may vary. On the basis of these evidences, we postulate that the elevation of IL1β & IL6 and its related downstream factors in HS and FCD lesions may contribute to the pathophysiology associated with epileptogenesis.

Changes in expression of miRNAs involved in regulating inflammatory responses have been observed in patients with HS, and relatively few reports are available in FCD^31,32^. Our results demonstrated differential expression of several microRNAs in HS and FCD. miR-204 play pivotal roles during epileptogenic process and STAT3 overexpression is found to be associated with decrease in miR-204^33^. Up regulation of STAT-3 might contribute to down regulation of miR-204-5 pin HS and FCD patients as observed in our study. Contradictory to our results, miR-21-5p is shown to be up regulated in children with MTLE and FCD^34,35^. However, down regulation of miR-21-5p is correlating with the up regulated IL1β and STAT-3 in the current study. Further studies are needed to extrapolate the clear association. miR-203-3p (upstream to IL 6, C-JUN, and CCR5) is found to be significantly decreased in FCD patients and unaltered in HS patients. There are few reports showing deregulation of its expression in MTLE tissues, thus, its contribution in regulating inflammatory processes needs further investigations^31^. Previous studies demonstrated that miR-223 forms a positive regulatory loop with IL6 for pro-inflammatory cytokine production^36^. Up regulated miR-155p expression is found to be associated with increased expression of pro-inflammatory cytokines and decreased expression of suppressor of cytokine signalling protein (SOCS1)^37^. Up regulation of IL6, IL1β, STAT-3 and miR-155p and down regulation of miR-223-3p are in sync with these published data. miR-Let-7a is shown to regulate anti-inflammatory factors like IL10 and IL4. Down regulation of IL10 and up regulation of IL6 in the present study, correlates well with the down regulated let-7a-5p^38^. We could not find significant differences in the levels of miR-106a-5p and let-7c-5p in MTLE and FCD patients as compared to controls, though changes in the expression of miR-106a-5p has been demonstrated by Kan *et al*.^31^ in MTLE patients.

Network analysis exhibited clustering of altered cytokines and downstream transcription factors in complex immunological networks. STAT-3, IL6, IL1β and C-JUN constituted major hubs showing complex interactions with various pathways i.e. JNK, MAPK, mTOR, EGFR, NF-κB, etc. Our results suggest that inflammatory processes might contribute to underlying neuronal-glia network dysfunctions. This analysis also supports the evidence that STAT-3 is the major downstream effector to the altered cytokine signalling cascade. Studies in rat model demonstrated that systemic administration of WP1066, a STAT-3 inhibitor, transiently reduce seizure-induced STAT3 *in vivo* and also lower the seizure frequencies over time^39^. Thus, STAT3 can be considered as a potential therapeutic target for both HS and FCD and needs further investigations.

### Chemokine-receptor-miRNA regulatory network

Two chemokines CCL3 and CCL4 are up regulated in both HS as well as FCD patients. Most studies so far report increased chemokine levels in human HS tissue or in experimental models for HS^5,18,28,40^. However, there is no report available regarding CCL3 and −4 expression in FCD so far. The effects of these chemokines are mediated by their G-protein-coupled receptors (GPCR’s), of which most important and widely studied is CCR5. We also found increased level of CCR5 in both HS and FCD patients. Increased CCL3/CCL4 signalling via CCR5 may relate to increased glial glutamate release and disruption of the BBB^18,40^.

A significant decrease in miR-204-5p (upstream to IL1β, CCR5) and miR-223-3p (upstream to IL6, CCL3) are demonstrated in both the pathologies. In addition, we observed decreased expression of miR-203-3p (upstream to IL 6, C-JUN, and CCR5) and miR-195 (upstream to CCL4) only in FCD patients. miR-223 is shown to directly target the chemoattractants CXCL2, CCL3, and the pro-inflammatory cytokine IL6^41^. However, no substantial evidence has been reported regarding miR-204 and miR-203 with reference to chemokine signalling. Decreased expression of miR-195 (upstream to CCL4) has been shown to up regulate RPS6KB1, (a known marker of mTOR activation)^42^. Interestingly, the mTOR pathway controls the function of dendritic cells (DCs) which regulates the chemokine production. Aberrant activation of mTOR pathway is also reported in FCD. Thus, miR-195 might be playing potential role in modulating chemokine activity and/or mTOR pathway in FCD. Recently, increased miR-155 is shown to be associated with increased production of the chemokines CCL3, CCL4, CCL5 and CCL8, and regulate chemokine receptor expression in rheumatoid arthritis patients^43^. So, the role of increased miR-155 expression in regulating chemokine signalling in epileptic cases cannot be ruled out. The current knowledge of the action of the semi RNAs with reference to chemokine signalling is not very much studied and demands further investigation.

Network analysis identified CCR5 as one of the major hubs showing complex interactions with various pathways i.e. JNK, MAPK, mTOR, EGFR, NF-κB, etc. CCR5 mediates cross talks with cytokine signalling to co-ordinate the release of many different cytokines/chemokines that are used by cells to orchestrate immune responses(Figs 3 and 4). CCR5 could be a potential therapeutic target in FCD and HS as both are network level disorders and aberrant activity of CCR5 could result in altered regulation of multiple immune responses.

### Differential pattern of cytokine/chemokine regulatory network

IL1β level is relatively higher in HS as compared to FCD, although it is not statistically significant. Chemokines, CCL3 and CCL4 levels are found to be relatively higher in FCD as compared to HS, however, it is not statistically significant. At the downstream expression level, only CCR5 (downstream target of CCL3 and CCL4) has been found to be significantly up regulated in FCD as compared to HS. At the microRNA level, expression of miR-204-5p and miR-195-5p are found be significantly down regulated in FCD as compared to HS. miR-204-5pand miR-195-5p are the possible microRNAs that could regulate the expression of chemokines and its receptors (CCR5) as performed with miRTarBASE^19^.

These apparent discrepancies are likely due to the different role that the activation of specific pro-inflammatory pathways may have on neuronal excitability, cell survival, and cellular and synaptic plasticity. Brain tissue from HS is shown to be mainly characterized by intrinsic inflammation involving activated microglia, parenchymal and perivascular astrocytes, scattered neurons, endothelial cells of microvessels and macrophages surrounding vessels and parenchyma. Cells of adaptive immunity, such as T cells and B lymphocytes, are shown to be scarce or absent^4^. In FCD, apart from intrinsic inflammation, the contribution of peripheral immune cells such as T cells and DCs, is shown to be more significant than in HS. DCs deserve attention in view of their ability to drive chronic inflammation interacting with T cells and regulating chemokine production^4,9^. It could be speculated that decreased expression of miR-195 may regulate the activity of mTOR signalling-denritic cell-chemokine regulatory axisand influencesthe production of chemokines in FCD^42,44^. As reported previously, relatively higher levels of IL1β in HS might be attributed to IL1β polymorphism in HS patients^45^.

Our study has several limitations. This is a preliminary study conducted on a set of inflammatory molecules, miRNAsand downstream transcription factors/receptor, selected based on their involvement in the pathogenesis of epilepsy, but it does not cover the whole potential network of molecules/pathways contributing to this disease. Another major limitation of this study is the small sample size which have not ageand sex matched cases vs controls. The amount of tissues resected in epilepsy and non-epileptic controls (tumor periphery) is so small in size that it limits the use of same tissues for multiple experiments including *in vitro* studies. In addition, due to several limitations associated with autopsy and trauma tissues, and better availability of tumor periphery tissues, we preferred to use histopathologically normal tumor periphery tissues as non-epileptic controls for this study. Hence, the mechanism and clinical implications of these pathology-specific immune alterations need to be clarified in a larger cohort of patients using different sets of non-epileptic controls. However, we believe that our preliminary study may contribute to understand the inflammatory networksinvolved in the pathogenesis of HS and FCD. Further investigations concerning the cytokine-chemokine network-mediated regulation of neuroinflammation may lead to novel therapeutic strategies against HS and FCD.

## Methods

### Tissue collection

This study was reviewed and approved by Institutional Ethics Committee (IEC), All India Institute of Medical Sciences (AIIMS), New Delhi, India and Institutional Human Ethics Committee (IHE), National Brain Research Centre (NBRC), Manesar, Haryana, India, before the study began. Informed consent was obtained from all the participants. All the experiments of this study were performed in accordance with guidelines of the IEC, AIIMS, New Delhi and institutional human ethics committee, NBRC, Manesar.

Patients with HS (twenty six) and FCD (twenty six) underwent phased pre-surgical assessment, and the pathology was demonstrated by documenting convergent data on MRI, video EEG (vEEG), fluoro-2-deoxyglucose positron emission tomography (FDG-PET) evaluations and electrocorticography (ECoG), and confirmed by histopathological examinations. Based on the concordant observations, decision for surgical resection was taken after explaining the available options and obtaining the written informed consent from the patient. There are no “ideal” or acceptable non-epileptic controls for this study which has been conducted on surgically resected tissue specimens from drug resistant epilepsy (HS and FCD) patients. Conceptually, the potential non-epileptic control tissue could be human brain samples resected from similar ages with non-seizure pathologies, such as tumors or trauma or post-mortem autopsy controls. Unfortunately, trauma is not very suitable as a control because within minutes of a traumatic impact, a robust inflammatory response is elicited in the injured brain^46^. In order to obtain useful information from post-mortem brain studies, it is important that the quality of the post-mortem brain samples meet to certain standards. Evidences exists indicating that RNA and protein degrades progressively with increasing post-mortem interval (PMI) and that measurement of gene expression in brain tissue with longer PMI may give artificially low values^47,48^. Recently, Roncon *et al*.^49^ have also raised doubt about using autopsy as a control for epilepsy surgery tissue in microRNA studies, suggesting that post-mortem modifications may have greater impact than the disease background on the data generated, with a potentially serious hindrance in their interpretation. Taken together, all these observations arises concerns on the use of autopsy tissue as control for this kind of studies. In addition, the potential role of systemic inflammatory diseases and infections may influence the cerebral inflammatory status^50^. Since this condition can represent a potential problem in the evaluation of the tissue content of cytokines/chemokines/mRNA and microRNA in autopsy samples, brain tissues resected because of associated tumor pathologies would be more ideal to be considered as a non-epileptic control. Tissue taken from the perimeter of the abnormal area are also used as non-epileptic controls in previous studies^2,23–25^. 22 cortical tissues obtained from the margins of tumors during surgical resection (a part of the planned surgical resection) in patients with brain tumors without any history of seizures have been included as non-epileptic control (Table 1). Detailed histological examination of the brain material from all patients used as control in this study showed that all samples were devoid of tumor tissue. Immediately after resection, for RNA based studies, tissues were collected in RNAlater solution, and for protein based studies, tissues were snap frozen in liquid nitrogen and stored at −80 °C until further use.

### Multiple inflammatory protein analysis by bead based multiplex immunoassay (MIA)

For this study, ten HS (H1–H10), ten FCD (F1–F10) and eight controls (C1–C8) were included. Protein was isolated using Cell lysis kit (Bio-rad) and estimated by bicinchoninic acid (BCA) Protein Assay Kit (Pierce, USA). MIA was performed on selected chemokines, cytokines and growth factors (IL1β, IL1Ra, IL6, IL10, CCL3, CCL4, TNFα, and VEGF). The experimental procedure was done using Biorad Bio-plex Pro assays kit as per manufacturer’s protocol. In brief, 50 µl of diluted bead solution and 50 µg of protein were added to each well in triplicate. Plate was incubated for 3 h and washed three times; afterwards, 50 µl of diluted biotin antibody was added to each well and incubated for 1 h. The plate was then washed and 50 µl of diluted streptavidin-PE was added to each well and incubated for 30 min. Finally, the plate was washed again and measured. Measurements and data analysis were performed using the Bio-Plex system in combination with the Bio-Plex Manager software version 4.1 (Bio-Rad Laboratories).

### Selection of downstream effectors and upstream miRNAs of altered inflammatory mediators

The known downstream effectors (STAT-3, C-JUN, ICER and CCR5) of altered cytokines/chemokines were selected from the available literature^13–18^. Regulatory miRNAs, miR-106a-5p, miR-223-3p, miR-21-5p, miR-195-5p, miR-204-5p, miR-203-3p, miR-155-5p, let-7a-5p and let-7c-5p were identified and selected through miRTarBase, the experimentally validated miRNA-target interactions database^19^.

### Gene expression analysis by quantitative real-time polymerase chain reaction (qPCR)

qPCR was performed to evaluate the expression level of downstream targets (STAT-3, C-JUN, ICER, and CCR5). HPRT (hypoxanthine phosphoribosyl-transferase) was included as reference gene^2^. Specific primers were designed using the Primer-BLAST (Primer3Input, version 0.4.0 and BLAST, available at http://www.ncbi.nlm.nih.gov/tools/primer-blast/) (Supplementary Table 1). qPCR was performed in an independent set of ten HS (H11–H20), ten FCD (F11–F20) patients and eight non-epileptic control samples (C9–C16). RNA was extracted, purified and analyzed for purity^2^. Purified RNA was reverse transcribed using high capacity cDNA reverse transcription kit (Thermoscientific) following the manufacturer protocol. Real time PCR amplifications were performed in CFX96 Real Time System (Bio-rad) with the following cycling parameters: an initial hot start of 95 °C for 3 min followed by 40 cycles of 95 °C for 5 s and 60 °C for 30 s. The 2−ΔΔCt method was used to quantify the relative normalized expression of studied genes. Melting curve analyses were performed to validate the specific generation of the expected PCR products.

### miRNA quantification by qPCR

Differential expression of selected upstream miRNAs was evaluated by qPCR using the QuantiMirSystem (SBI System Biosciences). Total RNA of tissues were extracted and purified using mirVana TM miRNA Isolation kit (Applied Biosystems/Ambion) following the manufacturer’s instructions. For this experiment six HS (H21-H26), six FCD (F21-F26), and six controls (C17-C22) were included. 200 ng of total RNA from each sample was reverse transcribed using the QuantiMir kit (System Biosciences (SMI), Mountain View, CA, USA) in a total volume of 20 μl.

mir16 was included as a normalization signal. The primer sequences used in RT-PCR were listed in Supplementary Table 1. cDNAs were mixed with specific forward primer and SYBR® Green Mastermix (Bio-Rad Laboratories,) plus the universal reverse primer. Real time PCR amplifications were performed in CFX96 Real Time PCR detection System (Bio-rad) with the following cycling parameters: 50 °C for 2 min and an initial hot start of 95 °C for 3 min followed by 40 cycles of 95 °C for 5 s and 60 °C for 30 s. The relative amount of each miRNAwas assessed using the comparative CT method normalized to miR16. Because even subtle changes in the miRNA levels could have profound effects on their mRNA targets, a fold-change cutoff of ≥1.3 (p < 0.05) was applied^51^. Melting curve analysis of the qPCR products verified product specificity.

### Gene network analysis

In order to assess the relationships between the inflammatory molecules, miRNAs and mRNAs of downstream targets which were altered in our study, gene network analysis was performed using Natural language processing-based (NLP) network discovery algorithms in gene spring software version 13.1.1. In brief, the software overlaid the list of significantly altered inflammatory molecules, miRNAs and mRNAs of downstream targets onto a global molecular network to derive networks in which the focus genes are projected as nodes and interacting partners are clustered around these nodes based on their reported connectivity. Gene relationship and interaction is extracted using NLP algorithms in Pathway Architect software available in Gene Spring ver. 13.1.1 (Agilent technologies). This tool extracts interactions for a given list of genes from Pubmed articles and Interact with text mining tools based on following relationships: binding, expression, metabolism, promoter binding, protein modification, regulation and transport etc. The network displays interactions among the entities and their neighbouring genes, including those that are not included in original list, thereby giving rise to nodes with different interrelationships^2,52^.

### Statistical analysis

Protein levels of inflammatory mediators are represented as median with range. mRNA and microRNA expression data are represented as ΔCt values with mean ± standard deviation (SD). Scatter diagram were plotted using GraphPadPrism softwareversion 7.03. One-way analysis of variance (ANOVA) followed by Dunnett’s test/Tukey’s post-hoc test is used as required to analyze the data between more than two group. A p-value of <0.05 is considered statistically significant. Sigma Plot softwareversion 13.0 (SYSTAT SOFTWARE INC) is used for statistical analyses.

### Ethical statement

We confirm that we have read the Journal’s position on issues involved in ethical publication and affirm that this report is consistent with those guidelines.

## Electronic supplementary material

Real time PCR primers used in this study
