# Supplementary material for: Comparative analysis of cytokine/chemokine regulatory networks in patients with hippocampal sclerosis (HS) and focal cortical dysplasia (FCD)
Source: Sci Rep. 2017 Nov 21;7:15904. doi: 10.1038/s41598-017-16041-w (PMC5698416; doi:10.1038/s41598-017-16041-w)
Supplement: Supplementary file 1 — Real time PCR primers used in this study [file 41598_2017_16041_MOESM1_ESM.pdf]

Title:

Comparative analysis of cytokine/chemokine regulatory networks in patients with hippocampal sclerosis (HS) and focal cortical dysplasia (FCD)

[1,2]Arpna Srivastava[#], [1,3]Aparna Banerjee Dixit[#], [1,2]Debasmita Paul, [1,4]Manjari Tripathi, [5]Chitra Sarkar, [1,2]P Sarat Chandra, [1,6]Jyotirmoy Banerjee*

[1]Centre of Excellence for Epilepsy, a joint collaboration of NBRC & AIIMS, New Delhi

[2]Department of Neurosurgery, All India Institute of Medical Sciences, New Delhi[3]

[3]Dr. B.R. Ambedkar Centre For Biomedical Research, University of Delhi, Delhi

[4]Department of Neurology, All India Institute of Medical Sciences, New Delhi

[5]Department of Pathology, All India Institute of Medical Sciences, New Delhi

[6]Department of Biophysics, All India Institute of Medical Sciences, New Delhi

[#]Contributed equally.

***Corresponding Author: Jyotirmoy Banerjee**

Department of Biophysics, All India Institute of Medical Sciences, New Delhi. Tel No.-+91-11-26549321. E-mail: jyotirmoybanerjee1@gmail.com

Table S1. Real time PCR primers used in this study.

Gene	Primers
STAT3	F - TCACATGCCACTTTGGTGTT
	R - CTTGCAGGAAGCGGCTATAC
C-JUN	F - TAACAGTGGGTGCCAACTCA
	R - TTTTTCTCTCCGTCGCAACT
ICER	F - TGCTACCATGGCAGTACCAA
	R - GATTGTTCCACCTTGGGCTA
CCR5	F - GGTTGGTGTTGCAGAAGGTT
	R - TGGTCTCCTTGCCCTAAATG
HPRT	F-GCTTTCCTTGGTCAGGCAGTA
	R-GGTCCTTTTCACCAGCAAGCT
miR-223a-3p	F- TGTCAGTTTGTCAAATACCCCA
miR-21-5p	F- TAGCTTATCAGACTGATGTTGA
miR-195-5p	F - TAGCAGCACAGAAATATTGGC
miR-204-5p	F- TTCCCTTTGTCATCCTATGCCT
miR-106a-5p	F- AAAAGTGCTTACAGTGCAGGTAG
miR-203-3p	F- GTGAAATGTTTAGGACCACTAG
miR-155-5p	F- TTAATGCTAATCGTGATAGGGGT
let-7a-5p	F- TGAGGTAGTAGGTTGTATAGTT

let-7c-5p	F-TGAGGTAGTAGGTTGTATGGTT
miR-16	F-TAGCAGCACGTAAATATTGGCG
